# A systematic review to compare physiotherapy treatment programmes for atraumatic shoulder instability

**DOI:** 10.1177/17585732221080730

**Published:** 2022-02-18

**Authors:** Jake Griffin, Anju Jaggi, Helena Daniell, Rachel Chester

**Affiliations:** 1 Bexley MSK, 8956 Oxleas NHS Foundation Trust, Erith and District Hospital, Erith, DA8 3EE, UK; 2 School of Health Sciences, Faculty of Medicine and Health Sciences, 6106 University of East Anglia, Norwich, Norfolk NR4 7TJ, UK; 3 Physiotherapy Department, 7597 Royal National Orthopaedic Hospital NHS Trust, Stanmore, London, UK; 4 Physiotherapy Department, 156671 Norfolk and Norwich University Hospital NHS Trust, Norwich, Norfolk, UK; 5 School of Health Sciences, Faculty of Medicine and Health Sciences, 6106 University of East Anglia, Norwich, Norfolk, NR4 7TJ, UK

**Keywords:** shoulder instability, physiotherapy, review, shoulder, outcome measures, atraumatic

## Abstract

**Background:**

Optimal physiotherapy treatment is uncertain for atraumatic shoulder instability (ASI), the primary aim of this systematic scoping review was to compare physiotherapy treatment programmes for people with ASI. The secondary aims were to evaluate outcome measures used and to compare the effectiveness of these programmes.

**Methods:**

CINAHL, EMBASE and Medline databases were searched for studies, except single case studies, published between 1950 and July 2021. 12 critical appraisal items covered three domains; internal validity, transferability to wider population and reporting.

**Results:**

Ten studies were included; one randomised controlled trial, 6 cohort studies and 3 case series. There were 491 participants. Treatment programmes included education, movement re-education, static posture correction, shoulder muscle strengthening, functional training, and adjuncts. All studies used patient reported outcome measures (PROMs), 7 of which reported a statistically significant improvement (*p* < 0.05) post-treatment. There was no clear relationship between programmes and outcomes. PROMs specific to shoulder instability were all found to detect statistically significant differences post-treatment.

**Discussion:**

There does not appear to be one optimal physiotherapy treatment programme for ASI. Future studies should use PROMs that are valid in the shoulder instability population and use more outcome measures that are specific to impairments being targeted.

## Introduction

Atraumatic shoulder instability (ASI) is characterised by abnormal movement or positioning of the humerus in the glenoid fossa leading to recurrent pain, subluxations, dislocations and functional impairment, in the absence of a history of significant preceding injury.^1^ The true prevalence of ASI is unknown but authors report incidence rates of between 4–10%.^1^ Most people will have underlying laxity with loss of muscle control resulting in symptoms. Some experience pathological laxity from repetitive micro-trauma, for example when using the arm at extremes such as throwing sports. Others experience congenital hyperlaxity where only a minor injury or change in demand results in symptoms.^1^

ASI is associated with reduced quality of life both physically^2^ and mentally.^3^ For some, changing position in bed^4^ and basic activities of daily living, for example reaching for a glass,^2^ can result in subluxation. They consequently begin to fear provocative shoulder movements and reduce their activity levels.^5^ This can result in decreased strength and joint position sense with concomitant increased pain and instability.^6^

The literature advocates a structured treatment programme to strengthen the muscles around the shoulder girdle, the majority reporting effective outcomes for between 50–80% of people.^1^ However, despite positive outcomes^1,7^ there is uncertainty regarding the optimal components of a treatment programme^7^ and why some may fail these approaches. This is compounded by the absence of a universally accepted classification system for shoulder instability^8–10^ and the presence of psycho-social factors contributing to the presentation.^11,12^

Several systematic reviews have evaluated the outcomes of physiotherapy management for ASI.^7,13,14^ However, none have compared treatments in detail, and four additional studies have been released since the most recent review.^15–18^ Given the relative infancy in this research area, an up-to-date review of the content of current treatment programmes will help to inform future clinical practice and research. The primary aim of this systematic scoping review was to describe the treatment programmes used in the literature for the management of ASI, the secondary aims were to evaluate the outcome measures used and where possible, to compare the effectiveness of these programmes.

## Methodology

This review was conducted and reported according to the Preferred Reporting Items for Systematic reviews and Meta-Analyses extension for Scoping Reviews.^19^

### Data sources and search

MEDLINE, EMBASE and CINAHL were searched for studies published in any language between 1950 and July 2021, using key words and MeSH headings related to condition (atraumatic instability), body region (shoulder) and intervention (rehabilitation/physiotherapy). The search strategy used in MEDLINE is presented in Table 1. Search terms were adapted for individual databases. Forward and backward citation tracking was also performed.

**Table 1.** Search strategy used in MEDLINE database.

#	Search term
1	((atraumatic OADJ3 shoulder) ADJ1 instabil*).ti,ab
2	(shoulder ADJ1 instabil*).ti,ab
3	exp *"JOINT INSTABILITY"/
4	exp *"SHOULDER JOINT"/
5	exp *"SHOULDER DISLOCATION"/
6	(atraumatic OADJ3 shoulder*).ti,ab
7	(multidirectional OADJ3 shoulder*).ti,ab
8	(multi-directional OADJ3 shoulder*).ti,ab
9	(1 OR 2 OR 3 OR 4 OR 5 OR 6 OR 7 OR 8)
10	*"PHYSICAL THERAPY MODALITIES"/ OR *REHABILITATION/
11	*"PHYSICAL THERAPY SPECIALTY"/
12	(physiother*).ti,ab
13	(10 OR 11 OR 12)
14	(9 AND 13)

### Eligibility criteria and study selection

The following study designs, published in full text peer reviewed journals, were eligible: case series, cohort studies, controlled clinical trials and randomised controlled trials (RCT). Eligible participants were of any age with ASI of any degree or duration, identified by a health practitioner. Data for groups consisting of more than 20% of participants with traumatic instability or previous surgery were excluded, as were studies of participants with traumatic brain injury, stroke and brachial plexus injury. Treatment must have been delivered by or involve one or more state registered (or equivalent) physiotherapists. Studies must have reported at least one patient-reported outcome measure (PROM).

Two independent reviewers (JG and HD) screened the titles and abstracts of all identified studies against pre-defined eligibility criteria, then retrieved and reviewed the full texts of those potentially eligible. Any disagreement on eligibility was resolved by the two reviewers through discussion.

### Data extraction, categorization and critical appraisal

Data were extracted by two independent reviewers (JG and HD). As several study designs were included, a custom designed critical appraisal tool was developed (see supplementary file 1), amalgamating published guidelines,^20–24^ and critical appraisal criteria used by previous reviewers.^7,25,26^

Extracted data criteria included study design, location, method of recruitment, inclusion and exclusion criteria, participant numbers and characteristics, patient-reported and impairment-based outcome measures, and results. Critical appraisal criteria were separated into three domains: reporting, transferability to the wider population and internal validity. Internal validity is the extent to which a causal link can be drawn between treatment programmes and their outcomes. Two reviewers (JG and HD) independently assessed the quality of each study using the critical appraisal tool. Any disagreement on the scoring and/or reasoning behind scoring was resolved through discussion. If no resolution was achieved this was to be discussed with the research team, however this was not required.

### Data synthesis

Treatment programmes are summarised in a table that details the intervention, its duration, and physiotherapy appointment length and frequency (Supplementary file 2). To facilitate ease of comparison, individual treatment components were identified, defined and tabulated by the clinical research team. These components were defined based on clinical transparency and designed to link directly to clinical practice and future research (Supplementary file 3).

## Results

### Study selection

**Figure 1.**
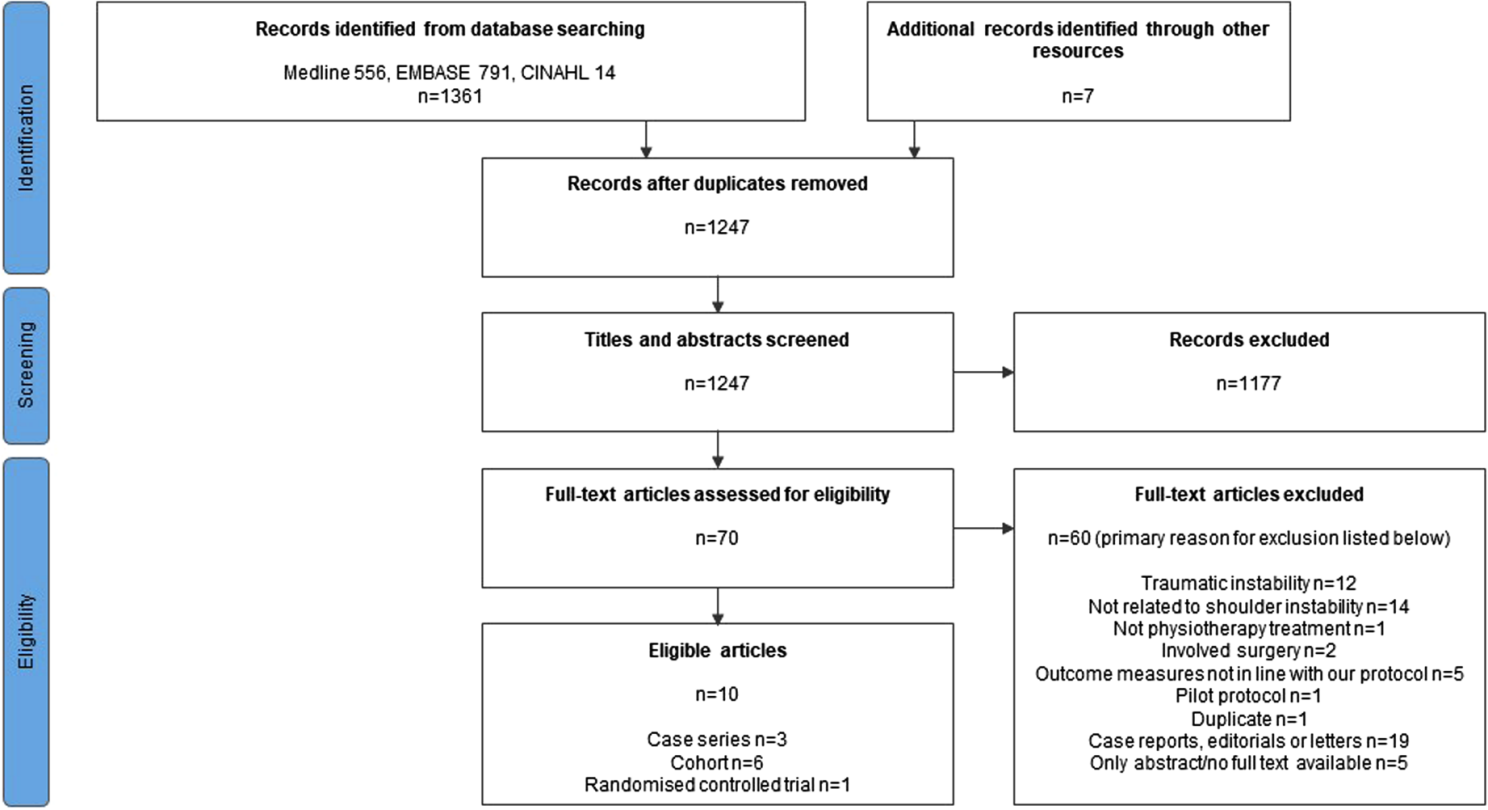
PRISMA flow diagram for study selection.

The results of the search strategy and study selection process are presented in the PRISMA flow diagram in Figure 1. After omitting duplicates, the number of initial records was 1247. Following title and abstract screening based upon the eligibility criteria above, 70 studies remained for full text screening, after which a further 60 studies were excluded. Of these, primary exclusion criteria were: Related to traumatic rather than atraumatic instability (*n* = 12), did not relate to shoulder instability (*n* = 14), did not relate to physiotherapy (*n* = 1), involved surgery (*n* = 2), included outcome measures that were not included in our protocol (*n* = 5), pilot protocols (*n* = 1), duplicates (*n* = 1), were case reports, editorials, or letters (*n* = 19), no full text available (*n* = 5). Ten studies remained. The search was initially performed in October 2019 and was updated in January and July 2021 with no new records found.

### Study characteristics

Three of the 10 eligible studies were case series, six were cohorts, and one an RCT, the latter comparing two treatment programmes.^17^ Five studies were based in the United Kingdom, two in Australia, and one in each of Japan, USA and Italy. Participants were recruited from hospitals in seven studies, sports medicine centres in two and a mixture of settings in one.

The number of participants receiving physiotherapy was approximately 491 ranging from 15^27^ to 85;^16^ one study reported the number of shoulders rather than participants.^28^ The mean age of participants was similar between studies, ranging from 16^29^ to 25 years.^28^ Proportions of female participants ranged from 53%^29^ to 80%.^17^ Mean duration of shoulder symptoms prior to physiotherapy was reported in eight studies and was over 12 months in all but one study.^27^ The greatest reported range in duration was one month to 21 years.^15^ Study design and participant characteristics are outlined in Table 2. Outcomes, as part of the data extraction form, can be found in Supplementary file 4.

**Table 2.** Study design and participant characteristics.

Study	Study design		Number of participants at start	Number at end	Mean age in years ± SD (range)	Total male/female (%)	Mean duration shoulder symptoms in months ± SD (range)
Bateman et al. (2019)	Cohort		66	51	21.65 (12–52)	23/43 (35/65%)	34 (1–252)
Blacknall et al. (2014)	Case series		19	19	16.1 ± 2.3 (13–22)	9/10 (47/53%)	17.3 (6–60)
Ide et al. (2003)	Cohort		46	46	20 (10–46)	12/34 (26/74%)	Not stated
Kiss et al. (2001)	Cohort		62 shoulders	62 shoulders	24.7 (both groups) (15–46.7)	37/24 (both groups) (61/39%)	Not stated
Merolla et al. (2014)	Cohort		15	15	19.1 ± 6.1	6/9 (40/60%)	6 (1–24)
Misamore et al. (2005)	Cohort		64	59	18.6 (13–34)	21/43 (33/67%)	Not stated
Scott et al. (2019)	Case series		85	85	Median 20 (9–46)	34/51 (40/60%)	Median 36 (1–17)
Takwale et al. (2000)	Cohort		50	50	17.3 (9–32)	21/29 (42/58%)	28 (4–168)
Warby et al. (2018)	Randomised controlled trial	W	18	17	21.8 (6.5, -)	3/15 (17/83%)	43.28 ± 87
		R	23	20	23 ± 6.5	5/18 (22/78%)	46.8 ± 45.8
Watson et al. (2018)	Case series		43	39	19.8 (12.5–31)	16/27 (37/63%)	38.2 (1–192)

SD; Standard Deviation, W; Watson MDI Program group, R; Rockwood Program group, %; Percentage.

### Critical appraisal of reporting, transferability to wider population, and internal validity

Critical appraisal was divided into the domains of reporting, transferability to wider population, and internal validity. It is outlined in Table 3.

**Table 3.** Study marks from custom critical appraisal tool.

Assessment criteria		Studies									
		Bateman et al. (2019)	Blacknall et al. (2014)	Ide et al. (2003)	Kiss et al. (2001)	Merolla et al. (2014)	Misamore et al. (2005)	Scott et al. (2019)	Takwale et al. (2000)	Warby et al. (2018)	Watson et al. (2018)
Reporting											
A	Clear and specific outline of programme aims	✓	x	x	x	✓	✓	✓	✓	✓	✓
B	Clear and detailed outline of components	✓	x	x	x	x	✓	x	✓	✓	✓
C	P values reported	✓	✓	x	x	✓	x	✓	x	✓	✓
D	Adverse events reported	x	x	x	x	✓	x	x	x	✓	x
E	Number of participants worsening reported	x	✓	x	✓	✓	x	x	✓	x	x
Transferability to wider population											
F	Reproducible selection criteria	✓	✓	✓	✓	✓	✓	✓	✓	✓	✓
G	Representative sample	x	x	x	x	x	x	x	x	x	x
Internal validity											
H	Standardised accurate outcome measures	✓	✓	✓	✓	✓	✓	✓	✓	✓	✓
I	Full PT attendance	✓	✓	✓	x	x	x	x	x	✓	✓
J	Adherence to home exercises	x	x	x	✓	x	✓	x	x	✓	x
K	No bias with loss to follow-up	✓	✓	✓	✓	✓	✓	✓	✓	✓	✓
L	Adequate adjustment for confounding	x	x	x	✓	x	✓	✓	x	x	x

### Reporting

#### Treatment programme descriptions

Three studies did not describe the aims of treatment in detail.^28–30^ Four studies provided detailed descriptions of exercises. One of these studies provided good detail in the text,^31^ another study detailed exercises by providing images, dosages and instructions for progression,^15^ and the two other studies^17,18^ cited previous papers outlining the programme in full.^32,33^

#### Presentation of results

Two studies systematically reported adverse events.^17,27^ One of these studies, the RCT, reported 14 minor adverse events; 5 in the Watson MDI programme and 9 in the Rockwood programme, which were *“all attributed to postexercise soreness and resolved with 48 h of rest and modification of exercise”*,^17^ [p.92]. This study also reported the incidence of dislocations, of which there were 8 in total during the treatment period (4 in the Watson MDI programme, 4 in the Rockwood programme). Neither study reported any serious adverse events with treatment. Four additional studies reported if participants’ conditions worsened with treatment;^27–29,34^ two of which reported no worsening,^29,34^ one reported that one participant *“had a poor clinical outcome requiring arthroscopic shoulder stabilization”*^27^ [p.268] and another study reported one shoulder to be worse after the programme but gave no further details.^28^

### Transferability to wider population

All studies provided reproducible inclusion and exclusion criteria. Participant selection was reported in 6 studies. There was also inconsistent reporting of participant selection, which appeared to be convenience sampling, consisting of patients attending clinics. It is unclear if all patients attending had an equal chance of being included in the studies. No study reported differences between consenters and non-consenters. By nature of those not having consented for inclusion their data is restricted, therefore such baseline comparisons are difficult to make.

### Internal validity

#### Physiotherapy attendance and adherence to home exercise programme

There was some ambiguity surrounding attendance to physiotherapy appointments and adherence to the home exercise programme. It was unclear if participants ‘completing the programme’ had attended all appointments and/or adhered to all exercises. Therefore, only unambiguous reporting is presented here. Five studies reported attendance to physiotherapy appointments,^15,17,18,29,30^ which ranged from 77%^15^ to 100%.^29,30^ Three studies reported adherence to home exercise programmes.^17,28,31^ Misamore et al.^31^ reported at 2 years *"all patients were initially compliant with their exercise program"*, but at the 8-year time point no patients were performing their exercise. Warby et al.^17^ observed significantly better adherence in the Rockwood group than the Watson group (adjusted between group difference, score 0–33, −2.5 (90% confidence interval −5.0 to −0.1)). Kiss et al.^28^ reported that 33 out of 59 participants were still carrying out their home exercises at follow-up (mean 3.7 years, ranging from 1–10 years), however these figures include some patients post-surgery.

#### Confounding factors

Two studies adjusted their outcomes for confounding factors.^16,28^ One provided an age and gender-adjusted Constant score.^28^ The other adjusted for several confounding factors.^16^

## Treatment programmes

There were nine different programmes from the ten studies. One study compared two programmes^17^ and there were two instances of the same programme being used in two studies; the Watson MDI programme^17,18^ and the programme in Kiss et al.^28^ and Blacknall et al.^29^ The Rockwood programme in Warby et al.^17^ is referenced as the original article published by Burkhead and Rockwood.^35^ The Watson MDI Programme in Warby et al.^17^ is also used in Watson et al.^18^ therefore this latter reference shall stand for both within this section. Programme descriptions can be found in Supplementary file 4.

### Treatment programme details

Programme durations were fixed in four studies,^17,18,20,30^ the remainder were dependent on participant progress. Two studies specified conditions for programme completion; return to sport or occupation^31^ and *“once the patient has an understanding of the problem, established a normal pattern of movement and can ‘feel the difference’”*^34^ [p.720]. Only three studies specified the length of physiotherapy appointments, all of which reported 30 min.^17,18,34^ The frequency of physiotherapy appointments ranged from three times a week in the first two weeks^27^ to once every 4–6 weeks,^31^ the former delivering the only programme that adjusted appointment frequency as the programme progressed; from 3x/week to 1–2x/week to once every four weeks.

### Treatment programme components

A consensus meeting was conducted to identify, define and categorise treatment components based on clinical transparency and utility. This was a complex process due to heterogeneous terms and descriptions between studies. Definitions can be found in Supplementary file 3. The resultant components were education, movement re-education, static posture correction, shoulder muscle strengthening, functional training, and adjuncts (Table 4). Table 4 includes the classifications of shoulder instability used by each study.

**Table 4.** Treatment programme components.

Study(s) Programme name Classification	Education	Functional training	Movement re-education	Static posture correction	Shoulder muscle strengthening (muscles targeted if specified in text)	Adjuncts
Bateman et al. ‘Derby Shoulder Instability Rehabilitation Programme' Atraumatic recurrent shoulder instability	✓	✓	✓		✓ Not stated	
Blacknall et al./Kiss et al. Anterior posterior shoulder instability/Multidirectional instability of the shoulder	✓	✓	✓		✓ Deltoid, periscapular muscles, rotator cuff	✓
Ide et al. Multidirectional shoulder instability			✓		✓ Rotator cuff, scapular stabilisers	✓
Merolla et al. Voluntary posterior instability			✓	✓	✓ Posterior glenohumeral and scapular muscles	
Misamore et al. Multidirectional shoulder instability	✓	✓	✓	✓	✓ Rotator cuff, scapular stabilisers	✓
Scott et al. Atraumatic shoulder instability	✓	✓	✓		✓ Not stated	
Takwale et al. Involuntary positional instability		✓	✓	✓	✓ Posterior muscle groups	✓
Warby et al./Watson et al. ‘Watson MDI Program' Multidirectional shoulder instability	✓	✓	✓	✓	✓ Deltoid, rotator cuff, scapular stabilisers	
Warby et al. ‘Rockwood Programme' Multidirectional shoulder instability	✓				✓ Deltoid, rotator cuff	

### Education

Six programmes explicitly described some form of patient education,^15,18,28,29,31^ only one of which did not report providing education on the nature of the condition.^31^ One programme included relative rest and advised participants to adjust activity levels to avoid causing undue pain.^31^ Two programmes approached education from a psychosocial perspective and emphasised the importance of self-management.^15,16^ Bateman et al.^15^ aimed to reduce the fear avoidance by advising that a degree of pain during exercise is normal and to be expected, and that pain does not equate to tissue damage. Scott et al. focused on participant capability rather than disability, using the analogy of a *“thoroughbred racehorse or a Ferrari […] needing some skilled handling to bring the best out”*^16^ [p.3].

### Movement re-education

Eight programmes included some form of movement re-education. Five programmes directly observed, corrected and educated participants on technical aspects of movement patterns^16,18,27–29,34^ with a focus on scapular and glenohumeral control. Four of these explicitly addressed static before dynamic control,^18,27–29,34^ whilst Scott et al.^16^ encouraged muscles to contract in a similar manner to the ‘normal’ side and focused on keeping the humeral head in-joint throughout movements within the pain-free ‘safe’ zone. All corrected technical aspects of movement patterns before starting shoulder muscle strengthening, and the milestone for progression to strengthening was the patient's ability to control their shoulder movement. The remaining three programmes adjusted strengthening exercises to facilitate movement re-education.^15,30,31^ Two aimed to improve speed of muscle activation^15,31^ and proprioception^15^ and the other aimed to induce *“synchrony training of scapulothoracic muscles”* with wall push-up exercises^30^ [p.343].

### Static posture correction

Four programmes corrected shoulder posture, including scapular position whilst maintaining a static glenohumeral joint.^18,27,31,34^ Merolla et al.^27^ also corrected trunk position. Merolla et al.^27^ and Takwale et al.^34^ aimed to reduce excessive internal rotation of the glenohumeral joint and avoid winging of the medial scapula border. The Watson MDI Programme aimed to develop scapula stability to centralise the humeral head by incorporating scapula setting against isometric resistance.^18^ Misamore et al.^31^ had participants initially perform scapula retraction, elevation and depression exercises *“without resistance simply to improve postural control of the scapula”*^31^ [p.467].

### Shoulder muscle strengthening

All nine programmes included shoulder strengthening exercises. Two programmes did not explicitly state which muscle groups were being targeted,^15,16^ however one of these did include descriptions and images with its exercises, allowing the reader to infer which muscle groups might be principally trained.^15^ The remaining seven programmes gave varying descriptions of similar body regions, for example, *“posterior muscle groups”*, *“scapulothoracic muscles”* and *“periscapular muscles”.* The rotator cuff was targeted in all these seven programmes. Other commonly targeted muscle groups were the deltoid and scapula stabilisers. See Table 4.

The recommended dosage for the strengthening exercises varied between, and at times within programmes. Progression of strengthening was achieved in several ways. Six programmes explicitly stated that resistance was progressed.^16,18,30,31,34,35^ Two programmes progressed from isometric to isotonic exercises.^30,31^ Three programmes progressed from banded isotonic exercises to isotonic exercises with weights such as dumbbells and pulley kits.^18,31,35^ Five programmes progressed exercises into increasing ranges of shoulder elevation.^16,18,27,31,34^ Progression was often judged on participants reaching milestones, such as reaching a target number of repetitions.^15,18,35^ Progression was also managed *“as condition of the shoulder allowed”*^31^ [p.467], and *“as per individual needs”*^16^ [p.4].

### Functional training

Six programmes included some form of functional training,^15,16,18,28,29,34^ which appeared to be performed towards the later stages. There were limited descriptions of functional exercises, apart from one programme that included images of all exercises.^15^

### Adjuncts

Four programmes used additional adjuncts.^28–31,34^ One programme used mirrors, closed circuit television, proprioceptive neuromuscular facilitation and biofeedback for the correction and retraining of scapulothoracic and glenohumeral movement patterns.^28,29^ A novel shoulder orthosis was used by one programme to increase scapular upward rotation and stability.^30^ Another programme used analgesics in its first stage.^31^ The fourth programme used a biofeedback machine in 14 shoulders and provided 7 out of the 50 participants with hydrotherapy.^34^

### Classifications

Different classifications had similar programme structure, and there were different structures for the same classification. Merolla et al.^27^ treated “*voluntary posterior instability”* and Takwale et al.^34^ treated “*involuntary positional instability”*, however both followed a similar structure from focusing on movement patterns, to scapular control, to strengthening and functional exercises. Programmes for multidirectional shoulder instability varied between including and excluding functional training, between technical and facilitated movement re-education, and strengthened different combinations of muscle groups.

## Outcome measures

All studies reported PROMs. Three PROMS were specific to shoulder instability;^36^ five studies reported on the Western Ontario Shoulder Index (WOSI), four on the Oxford Shoulder Instability Score (OSIS), and two the Melbourne Instability Shoulder Score (MISS). One study reported on the Disability of the Arm, Shoulder and Hand score (DASH),^27^ one reported on Shoulder Pain and Disability Index (SPADI)^27^ and one on the Orebro Musculoskeletal Pain Questionnaire (OMPQ).^17^ All reported a statistically significant improvement (*p* < 0.001) in these PROMs between pre- and post-treatment, aside from the OMPQ where no within-group statistical analysis was performed. The magnitude of difference pre- to post-treatment varied considerably and may in part reflect the different end points at which outcomes were collected, with most end points collected within six months. Up to six months follow up, improvements in the WOSI ranged from a mean of 37.2% (SD 11.3)^29^ to 94.46% (SD 15.7).^17^ For the OSIS, pre- to post-treatment improvement ranged from a mean of 42.2% (SD 6.97)^18^ to 80.18% (SD 6.7).^29^ For the MISS, pre- to post-treatment improvement ranged from a mean of 36.76% (SD 21.4)^17^ to 65.55% (SD 13.1).^17^ A mean improvement of 33.08% (SD 7) was reported for the DASH,^27^ and 36.5% (SD 24.7) for the two groups’ OMPQ outcomes by Warby et al.^17^ SPADI values were not reported at all timepoints.^27^ One study explicitly looked at longer term outcome,^27^ where at the two-year follow-up, a mean improvement of 86.35% (SD 3) was reported for the DASH.

Impairment-based outcomes were reported in a small proportion of studies. Although strengthening was a key component of rehabilitation in all 10 studies, only three reported strength-based measures.^17,18,30^ These included strength of various muscle groups in various ranges, for example external rotation at 0 degrees abduction. Significant improvements were found post-treatment (*p* < 0.05) for all those where within-group statistical analysis was performed. Watson et al.^18^ found the greatest percentage improvements in strength from baseline, with up to 50.9% and 52.4% respectively in internal and external rotation at 90 degrees abduction.

Three studies reported range of motion^17,18,27^ but used different measures. Merolla et al.^27^ reported significant improvements in flexion, abduction and external rotation ranges at all time points, but no change in internal rotation at any time point. External rotation showed the greatest mean improvement of 125% (SD 6). Watson et al.^18^ reported significantly greater scapular upward rotation post-treatment, between rest and 60 degrees glenohumeral abduction. Warby et al.^17^ reported greater scapular upward rotation at all angles of abduction but did not perform within-group statistical analysis. Changes in scapular upward rotation at rest ranged between a mean of 132.06% (SD 5.5)^18^ and 183.33% (SD 6.4).^17^ Changes in scapular upward rotation at 90 degrees glenohumeral abduction ranged between a mean of 2.39% (SD 8.4)^18^ to 25.68% (SD 12.2).^17^ Changes in scapular upward rotation at end of range glenohumeral abduction ranged between a mean of −2.61% (SD 5.7)^18^ and 9.46% (SD 6.6).^17^

The RCT by Warby et al.^17^ reports no statistically significant difference in outcomes between the Watson and Rockwood rehabilitation programmes at 6 week follow up. Statistically significant between-group differences favouring the Watson^32,33^ compared to the Rockwood^35^ programmes were reported at longer term follow up. This was despite the reduced compliance in the Watson group (-2.5; 95% CI, −5.0 to −0.1; *P* = 0.042). Effect sizes were large for PROMS specific to shoulder instability, at 12 weeks for the WOSI (ES 11.1; 95% Confidence interval [95% CI], 1.9 to 20.2, *p* = 0.18) but not the MISS (ES: 8.8; 95% CI 20.5 to 18.2, *p* = 0.64), and at 24 weeks for both the WOSI (ES: 12.6; 95% CI, 3.4 to 21.9; *p* = 0.008) and the MISS (ES:15.4; 95% CI, 5.9 to 24.8; *p* = 0.002). Apart from flexion strength, range of abduction and pain scores at 24 months, there were no significant differences between groups in the OMPQ, pain scores, incidence of dislocation, global rating of change or satisfaction scores. This study's results suggest that some meaningful clinical changes are only detected by outcome measures specific to shoulder instability.

Three studies reported on prognostic factors associated with outcome and provided sometimes contradictory evidence.^16,28,31^ In one study,^16^ posterior instability and early referral to physiotherapy were associated with a better outcome but previous surgery, age, duration of treatment and previous physiotherapy had no significant association. However, another study reported that sex, age, previous surgery, psychological problems, and the direction of instability did have significant effects on the final objective or subjective outcome.^28^ Another study reported that unilateral involvement, higher grades of laxity, and difficulties performing daily activities were predictive of surgical treatment.^31^ Further research is needed in theranostic factors specific to this cohort and whether they differ from other shoulder and musculoskeletal conditions.

## Discussion

### Summary of main findings

This review is the first to define and compare the components of published physiotherapy treatment programmes and the first to examine the outcome measures used in the literature.

From the ten studies included in this review, nine treatment programmes were described, with two programmes being investigated in multiple studies. We identified and defined six treatment components used in different combinations across the included programmes. These were: education (used in six programmes), movement re-education (eight), static posture correction (four), shoulder muscle strengthening (all nine), functional training (six), and adjuncts (four). Programme duration and frequency of sessions varied; some were fixed, and others were dependent on participant progress. Relationships between programmes and outcomes were unclear, as only one study compared the effectiveness of two programmes.

Seven studies reported a statistically significant improvement in PROMs between pre- and post-treatment, ^15–18,27,29,30^ the remaining three either did not report baseline values or did not report statistical significance.^28,31,34^ Four studies reported impairment-based outcome measures,^17,18,27,30^ reporting statistically significant improvements for strength and range of motion variables.

Only one study, an RCT, compared different programmes.^17^ The Rockwood programme only included two components: education and shoulder muscle strengthening. The Watson MDI Programme used functional training, movement re-education and static posture correction in addition to education and shoulder muscle strengthening. Significantly improved outcomes were reported following the Watson MDI programme when compared with the Rockwood programme. Interestingly though, compliance was lower in the Watson group. Over the remaining nine studies there was no clear trend towards better outcomes if a programme used more, fewer, or particular components.

There was no relationship between classifications, direction of instability and programmes. Different classifications had similar programme structure, and there were different structures for the same classification. This suggests that current programmes are not bespoke to classes of ASI.

PROMs specific to shoulder instability were all found to detect statistically significant differences between pre- and post-treatment. This was not always the case for other PROMs. While all studies included some form of strengthening, only three studies reported strength-based measures.

### Comparison with other reviews

Three previous systematic reviews have investigated the conservative management of various forms of ASI.^7,13,14^ Each of these reviews commented that the low quality of included studies limited the strength of any recommendations. Warby et al.^7^ and McIntyre et al.^14^ called for more high quality RCTs. No scoping reviews have been performed to date. This systematic scoping review is the first to define and compare components of published physiotherapy treatment programmes and the first to examine the outcome measures used in the literature.

The descriptions of the programmes varied in clarity and detail. Clarity is an issue when different studies use several seemingly interchangeable terms that describe the same concept. For instance, under the umbrella of movement re-education, *“dynamic postural control”*, *“motor control”*, *“muscle patterns”*, and *“movement patterns” are used. Varied detail is evident when comparing the short description by Ide* et al.^30^ *with the more exhaustive description by Bateman* et al.*.*^15^ *Warby* et al.^7^ *concluded that they could not recommend a single programme due to the lack of detailed descriptions. The same applies to this current review.*

### Recommendations for clinical practice

Muscle strengthening is the predominant feature of ASI treatment programmes, and it appears from this review that no one specific programme structure is superior to another. This implies if treatment includes education, movement re-education, functional training, posture correction and strengthening, patients should improve. However, some patients are resistant to such regimes. Such patients may fear provocative shoulder movements and therefore reduce their engagement in meaningful activities.^5^ In addition, they may suffer from mental health problems such as anxiety and depression, which can affect pain levels, adherence and motivation in therapeutic engagement.^12^

The current authors would therefore recommend addressing these psychosocial factors when attempting to implement physical programmes of exercise. This approach is used in the two most recent programmes by Bateman et al.^15^ and Scott et al..^16^ They aimed to reduce fear of pain, emphasised patient capabilities, and underlined the importance of self-management of the condition. Addressing psychosocial factors through education is argued to be a pillar of self-management promotion^37^ and may help to reduce fear of movement that is seen in those with shoulder dislocations.^38^

Those with ASI may benefit from improved body and limb awareness when training with video camera feedback, or proprioceptive feedback in the form of tape or biofeedback. These methods were classed as adjuncts. Outcomes improved with simple adjuncts such as mirrors and proprioceptive neuromuscular facilitation to assist in correcting movement patterns and improving body awareness. Improved outcomes were also found when treatment was combined with a shoulder orthosis that increased scapular inclination and stability.

Common postural issues found in this population include winging of the medial scapula border and a humeral head that is not centralised in the glenoid cavity. It may therefore be beneficial to include some form of postural correction. This may involve improving scapula stability through isometric resistance, or improving dynamic scapula control with isotonic retraction, elevation and depression exercises.

In the included programmes there were two main approaches to movement re-education; one focused on technical aspects of movement patterns and the other adjusted strengthening exercises to facilitate movement re-education. Bateman et al.^15^ argue that patients who over-medicalize and focus too closely on their shoulder may benefit from ignoring technical aspects of movement patterns and instead focus on strengthening exercises to gain stability and function.

Muscle strengthening exercises should be included in such a programme. Muscle groups that appear to be the most important to target are the rotator cuff, deltoid and scapula stabilisers. Progression of these exercises should be patient-specific; *“as condition of the shoulder allowed”*^31^ and *“as per individual needs”.*^16^

Adherence to a programme may be affected by its acceptability and factors unrelated to physiotherapy treatment. In the study by Warby et al.^17^ the Rockwood group had significantly better adherence than the Watson group. Interestingly, as better outcomes were reported in the Watson group, this may suggest that adherence is less influential for short-term outcomes than programme content. There was good adherence reported in the study by Kiss et al.^28^ where at follow-up, on average 3.7 years after commencing the programme, 33 out of 59 patients were still carrying out their exercises. One reason for this may be the input from occupational therapy in addition to the home exercise programme, something that was not evident in the other programmes. Adherence is an important individual factor for this long-term condition if patients are to continue to experience ongoing benefits from their programmes.

### Recommendations for future research

Treatment programmes in future studies should provide more consistent detail and clarity, to maximise reproducibility in future research. This should include a definition of ASI. In addition, more long-term follow-up is required to match the nature of this long-term condition.

Future studies should use PROMs that are valid in the shoulder instability population. These outcome measures are specific to tasks and positions often associated with instability. They can at times be the only outcomes that show statistically significant differences. Future studies should also use more outcome measures that are specific to the impairments being targeted. Such outcomes could include strength-based measures. This will improve our understanding of the mechanisms by which treatments are effective.

With limited understanding of proposed mechanisms behind improvements in outcome, using a mixed methods research design may be a useful way of illuminating how or why programmes are successful or unsuccessful for participants.^39^ Mixed methods research captures the complexity of patients’ experiences, gives insight into the diversity of perspectives^40^ and, importantly, puts focus on the person being treated.

Further recommendations could include a consensus study to provide i) a universal definition of ASI and ii) recommendations for outcome measures, prior to further research investigating the effectiveness of integrating a psychological approach to existing education and exercise.

## Conclusion

There does not appear to be one single most effective physiotherapy treatment programme for ASI. Presentation within the population can vary in several domains including pathoanatomically and psychologically,^12^ in a similar manner to the variation in low back pain.^41^ Physiotherapists should therefore appreciate this variance and complexity when drawing from research evidence to inform treatment choice for this population.^42^ Instead of following one fixed programme, physiotherapists should use clinical reasoning to build a programme that combines applicable concepts from a number of sources to suit a given individual.

## Supplemental Material

sj-docx-1-sel-10.1177_17585732221080730 - Supplemental material for A systematic review to compare physiotherapy treatment programmes for atraumatic shoulder instability Click here for additional data file. Supplemental material, sj-docx-1-sel-10.1177_17585732221080730 for A systematic review to compare physiotherapy treatment programmes for atraumatic shoulder instability by Jake Griffin, Anju Jaggi, Helena Daniell and Rachel Chester in Shoulder & Elbow

sj-docx-2-sel-10.1177_17585732221080730 - Supplemental material for A systematic review to compare physiotherapy treatment programmes for atraumatic shoulder instability Click here for additional data file. Supplemental material, sj-docx-2-sel-10.1177_17585732221080730 for A systematic review to compare physiotherapy treatment programmes for atraumatic shoulder instability by Jake Griffin, Anju Jaggi, Helena Daniell and Rachel Chester in Shoulder & Elbow

sj-docx-3-sel-10.1177_17585732221080730 - Supplemental material for A systematic review to compare physiotherapy treatment programmes for atraumatic shoulder instability Click here for additional data file. Supplemental material, sj-docx-3-sel-10.1177_17585732221080730 for A systematic review to compare physiotherapy treatment programmes for atraumatic shoulder instability by Jake Griffin, Anju Jaggi, Helena Daniell and Rachel Chester in Shoulder & Elbow

sj-docx-4-sel-10.1177_17585732221080730 - Supplemental material for A systematic review to compare physiotherapy treatment programmes for atraumatic shoulder instability Click here for additional data file. Supplemental material, sj-docx-4-sel-10.1177_17585732221080730 for A systematic review to compare physiotherapy treatment programmes for atraumatic shoulder instability by Jake Griffin, Anju Jaggi, Helena Daniell and Rachel Chester in Shoulder & Elbow
