# Supplementary material for: A systematic review to compare physiotherapy treatment programmes for atraumatic shoulder instability
Source: Shoulder Elbow. 2022 Feb 18;15(4):448–60. doi: 10.1177/17585732221080730 (PMC10395403; doi:10.1177/17585732221080730)
Supplement: sj-docx-1-sel-10.1177_17585732221080730 - Supplemental material for A systematic review to compare physiotherapy treatment programmes for atraumatic shoulder instability [file sj-docx-1-sel-10.1177_17585732221080730.docx]

| **Supplementary material** 1 Custom critical appraisal tool | | | | | | | | | |
| --- | --- | --- | --- | --- | --- | --- | --- | --- | --- |
| Criteria Code | Description |  | Definition |  | Marking Options |  | Mark Given |  | Source |
| Reporting | | | | | | | | | |
| A | Clear and specific outline of treatment programme aims |  | Overall aims should be explicitly outlined. If this is unclear, or ambiguous this should be reported as No. |  | Yes (✓), No (x) |  |  |  |  |
| B | Clear and detailed outline of treatment programme components |  | Treatment programme components might include exercises, education, adjuncts etc. A clear outline relates to describing what exercises were performed and how, the form of education implemented, how the adjunct was used, etc. If this is not the case in the publication, but a clear and detailed outline of the treatment programme is referenced elsewhere, this should be answered Yes |  | Yes (✓), No (x) |  |  |  | Informed by: Warby et al. 2014 appendix b |
| C | Actual probability values reported for main outcome(s) irrespective of their value, except where ≤ 0.001 |  | Were probability values reported as actual values i.e. 0.035 instead of p<0.05? |  | Yes (✓), No (x) |  |  |  | Main source: Downs and Black 1998 qu. 10 (with additional comments from author). |
| D | Adverse events reported or acknowledged |  | This should be answered yes if the study demonstrates that there was a comprehensive attempt to measure adverse events. |  | Yes (✓), No (x) |  |  |  | Source: Downs and Black 1998 qu. 8 |
| E | No. of participants worsening with PT reported |  | This should be answered yes if the study states the number or proportion of participants whose outcome got worse during treatment. |  | Yes (✓), No (x) |  |  |  |  |
| Transferability to wider population | | | | | | | | | |
| F | Reproducible selection criteria |  | In cohort studies and trials, inclusion and exclusion criteria should be given such that findings can clearly and unambiguously be applied to specific patients in clinical practice. |  | Yes (✓), No (x) |  |  |  | Informed by: Downs and Black 1998 qu. 3; Hayden 2006 domain 2; Verhagen 1998 qu 1; Kuijpers et al 2004 criteria B |
| G | The study sample represents the exposed population from which they were recruited |  | The study must identify the source population for patients and describe how the patients were selected. All appropriate patients from the source population that fulfil the eligibility criteria should have an equal chance of being invited onto the study. Validation that the sample was representative would include demonstrating that the distribution of the main confounding variables was the same in the study sample and the source population. Where presentation concentrates on eligibility and does not record (potential) differences between consenters and non-consenters this should be recorded as No. |  | Yes (✓), No/Unable to determine (x) |  |  |  | Main source: Downs and Black 1998 qu. 12 (with additional comments from Rachel Chester 2013). Also informed by: Hayden 2006 domain 3, Wells 2011 “Selection” qu 1 and 2 |
| Internal validity | | | | | | | | | |
| H | Standardised accurate outcome measure |  | This should be answered Yes where outcome measures are clearly described and reproducible, without ambiguity of measures or categories. This includes when references are made to other studies which have demonstrated the accuracy of the outcome measure. |  | Yes (✓), No/Unable to determine (x) |  |  |  | Informed by: Downs and Black 1998 qu. 20; Hayden 2006 domain 9; Kuijpers et al 2004 criteria K |
| I | Full PT attendance |  | Was the proportion of participants completing the full course of physiotherapy stated? |  | Yes (✓), No (x) |  |  |  |  |
| J | Adherence with home exercises |  | Was the proportion of participants completing their prescribed home exercises reported? This should only be answered Yes if actual results are presented. Studies which state that exercise diaries were provided and/or compliance monitored but do not provide results, should be answered unable to determine |  | Yes (✓), No/Unable to determine (x) |  |  |  |  |
| K | Loss to follow up for final outcome measure unlikely to introduce bias |  | If loss to follow up is not reported, unclear or ambiguous this should be reported as No. State loss to follow up in percentage. If the proportion lost to follow up unlikely to introduce bias - percentage lost to follow up less than or equal to 20% or not due to factors that are prognostic for outcome - this should be reported as Yes. |  | Yes (✓), No/Not applicable (x) |  |  |  | Informed by: Downs and Black 1998 qu. 26; Hayden 2006 domains 4 & 5, Kuijpers et al 2004 criteria I, Wells 2011 “outcome qu 3”, ROBINS-I 1.3 |
| L | Adequate adjustment for other confounding prognostic factors |  | This should be reported as no for trials if: the main conclusions of the study were based on analyses of treatment rather than intention to treat; the distribution of known confounders in the different treatment groups was not described; or the distribution of known confounders differed between the treatment groups but was not taken into account in the analyses. In nonrandomised studies if the effect of the main confounders was not investigated or confounding was demonstrated but no adjustment was made in the final analyses the question should be answered as no. If unclear, or ambiguous this should be reported as No. Where only univariable analysis has taken place a clinical judgement may be required to assess whether other factors should have been considered as potential confounders. |  | Yes (✓), No (x) |  |  |  | Informed by: Downs and Black 1998 qu. 25; Hayden 2006 domain 11 |
