# Supplementary material for: A systematic review to compare physiotherapy treatment programmes for atraumatic shoulder instability
Source: Shoulder Elbow. 2022 Feb 18;15(4):448–60. doi: 10.1177/17585732221080730 (PMC10395403; doi:10.1177/17585732221080730)
Supplement: sj-docx-2-sel-10.1177_17585732221080730 - Supplemental material for A systematic review to compare physiotherapy treatment programmes for atraumatic shoulder instability [file sj-docx-2-sel-10.1177_17585732221080730.docx]

| **Supplementary material 2** Treatment programmes | | | |
| --- | --- | --- | --- |
| Study(s) Programme name | Description of intervention | Duration of intervention | Number of times participants saw physiotherapist/Length of appointments |
| Bateman et al. (2019) Derby Shoulder Instability Rehabilitation Programme' | Derby Shoulder Instability Rehabilitation Programme. Progressive exercises working on speed of muscle activation, plyometrics and deceleration of fast movement (Section 1 - max reps to fatigue or target reps, 2x/day), proprioception, muscle balance and trunk stability (Section 2 - 5 reps of patient's maximum ability or specified target time, 2x/day). Education on condition. | 30 weeks (range 6-51 weeks) | 6.9 (mean)/Not stated |
| Blacknall et al. (2014)/Kiss et al. (2001) | Explanation of the condition to the patient. Proprioceptive input to improve joint position sense, and re-learning correct movement patterns with development of strength and endurance in the scapulothoracic and glenohumeral muscles. Mirrors, closed circuit television, proprioceptive neuromuscular facilitation, and biofeedback used for the correction and retraining of the scapulothoracic and glenohumeral movement patterns. Improvement of muscle balance and proprioception using strengthening exercises, closed chain exercises, and stamina training. Patients instructed on how often the exercises had to be performed each day. Occupational therapy and a home exercise programme used to promote and to maintain the functional capacity of the shoulder. | 172 days (SD 165, range 1-680) / Not stated | Not stated/Not stated |
| Ide et al. (2003) | Strengthening rotator muscles and scapula stabilizers. Isometric (IR, ER, 8 seconds on, 2 seconds off) and isotonic (Thera-band) exercises, according to Burkhead and Rockwood. Wall push-up exercises. Performed daily with novel shoulder orthosis - increased scapular inclination and stability | 8 weeks | Not stated/Not stated |
| Merolla et al. (2014) | Three phases. (1) assessment and correction of abnormal muscles patterns and faulty posture - to prevent posterior dislocation during active shoulder flexion. (2) restoration of physiological active scapular motion - trained to perform correct scapular movement during arm elevation according to the plane of movement. Instructed to perform the flexion exercises with scapular control at home initially with arm in neutral abduction to activate external rotators and posterior deltoid, inhibiting internal rotators. Progressed to increasing abduction and flexion >90 degrees. When glenohumeral joint was stable through full range of motion progressed to (3) muscle training and strengthening - strengthening scapular muscles (rhomboids, lower and middle trapezius, serratus anterior) and posterior glenohumeral muscles (posterior deltoid and external rotators) with elastic bands and high number of repetitions | 12 months | First 2 weeks - 3x/week; third week - 1 or 2x/week depending on proficiency in performing exercises; home rehabilitation - once every 4 weeks/Not stated |
| Misamore et al. (2005) | Home exercise program. Goals - improve strength, stamina and coordination of shoulder muscles, to improve dynamic control of the glenohumeral joint. Similar exercise program to Burkhead and Rockwood (1992). Phase I - relative rest from provocative activities, analgesics, gentle range-of-motion exercises to reduce pain. Phase II once pain subsided - daily rotator cuff (internal and external rotation, flexion, extension, abduction - with arm in more abduction and at greater contraction speeds as exercises progressed) and parascapular muscle strengthening exercises (retraction, elevation, depression of scapula - with elastic tubing and arm weights as exercises progressed), slowly progressive strengthening, condition of shoulder and response to exercise determined aggressiveness and progression of exercises. Aimed for 15-20min 3x per day, relatively pain-free. Advanced exercises included rubber tubing with progressed resistance, dumbbell weights. Push ups performed as tolerated. Phase III - sports-specific exercises. Phase IV - returning to sports or work. Patients were encouraged to continue their home exercise program indefinitely | Until symptoms improved or condition reached a plateau | Every 4-6 weeks/Not stated |
| Scott et al. (2019) | Educate - Discuss with Patient family to understand management of atraumatic instability. Mobilise - Unrestricted active movement of thoracoscapulo-humeral system; Topical heat, massage, but predominantly selfstretch; As tolerated by patient. Activate - Able to produce force in thoraco-scapulohumeral system without subluxation/pain; Predominantly isometric exercises in safe zones; Aiming for 10 reps, 10 second holds, 4 times daily; Adapt to patient convenience and response. Control - Able to move arm freely, with moderate load and without subluxation/pain; Predominantly dynamic exercises; Aiming for 10 reps, 4 times daily; Adapt to patient convenience and response. Perform - Full function without subluxation/pain; Bespoke exercises; Build up to patient requirements | 17 weeks (range 4-129) | Not stated/Not stated |
| Takwale et al. (2000) | Two phases. (1) visual analysis of abnormal muscle patterns. Careful observation of the resting posture and the movement which occurs as elevation of the shoulder is initiated. (2) regain independent scapular movements of elevation, depression, protraction and retraction. 3-4 treatment sessions on each of the first two days. Regain active control of the scapula so that the glenoid remains correctly aligned with the head of the humerus during shoulder elevation. The patient works on the glenohumeral movement which opposes the primary abnormal movement pattern. Shoulder in neutral abduction>elevation in the scapular plane and true abduction>movement with increasing complexity. Activities done with the shoulder flexed forward and abducted to 90°. Functional activities. Resisted weights such as lateral pull-downs, push-ups and any activities which work the posterior muscle groups. Low weights and high repetitions. Hydrotherapy used as a supplement in seven patients. Once the patient has an understanding of the problem, established a normal pattern of movement and can ‘feel the difference’, they are discharged. Continue to practise the patterns of movement on a daily basis. Reviewed at three weeks. Correct any return to abnormal patterns of movement. | No time limit imposed, based on patient's own progression. Reviewed 3 weeks post-discharge | Inpatients - one night in minimum care unit and 1.4 additional visits. Outpatients - 4.5 visits/Inpatients - not stated. Outpatients - approx 30 minutes |
| Warby et al. (2018)/Watson et al. (2018) 'Watson MDI Program' | Watson MDI programme: home-based exercises. Education regarding nature of injuries, rationale for exercise treatment, importance of compliance to the programme. Assessment determines what scapular and humeral head position the participant will need to retrain and maintain throughout the intervention. Retraining and maintaining good scapula and humeral head motor control prior to rotator cuff and deltoid strengthening. Load was applied to each exercise with the weight of the participant's arm, a Theraband, or a dumbbell. Recruitment dose initially, followed by endurance dose, then strengthening/hypertrophy dose. Final stages incorporated functional and/or sport-specific exercises | 12 weeks | 12 (1x/week)/30 min |
| Warby et al. (2018) 'Rockwood Programme' | Rockwood Instability Programme: home-based exercises. Education regarding nature of injuries, rationale for exercise treatment, importance of compliance to the programme. Concurrently strengthening all 3 parts of the deltoid and internal and external rotators of the glenohumeral joint in 2 phases. Phase 1 - 5 exercises for the roator cuff and deltoid with 6 progressive levels of Theraband resistance. Phase 2 - Began when participant progressed through all resistance bands in phase 1, perform same exercises as phase 1 using 4kg weight with pulley kit | 12 weeks | 12 (1x/week)/30 min |
