# Supplementary material for: A systematic review to compare physiotherapy treatment programmes for atraumatic shoulder instability
Source: Shoulder Elbow. 2022 Feb 18;15(4):448–60. doi: 10.1177/17585732221080730 (PMC10395403; doi:10.1177/17585732221080730)
Supplement: sj-docx-3-sel-10.1177_17585732221080730 - Supplemental material for A systematic review to compare physiotherapy treatment programmes for atraumatic shoulder instability [file sj-docx-3-sel-10.1177_17585732221080730.docx]

**Supplementary Material 3 – Component Definitions**

**Adjuncts**

Supplementary to other elements and goals of the treatment programme. Used in addition to main elements in an aim to maximise effectiveness.

Adjuncts in treatment programmes included in the current review:

Analgesics

Biofeedback

Hydrotherapy

Mirrors

Orthosis

Proprioceptive neuromuscular facilitation

Video camera feedback

**Education**

Exchanging information with the patient in order to improve their health behaviours relating to the condition.

Where defined in text

Informed about the condition, including pain expectations and prognosis

Explaining the importance of adherence to the treatment programme

Encouraging and fostering self-management, including use of relative rest, addressing pain, sleeping position

**Functional training**

Rehabilitation that mimics and prepares the patient for activities of daily living.

Where defined in text

Terms and phrases that signify functional training in treatment programmes included in the current review:

Functional daily activity

Functional positions and activities

Individual needs

Occupational/recreational/sporting demands

Return to sports/work

Sport-specific exercises

**Movement re-education**

Rehabilitation focusing on quality of movement produced by the shoulder. This may be facilitated by addressing other joints in the kinetic chain.

Where defined in text

Terms and phrases that signify movement re-education in treatment programmes included in the current review:

Active/dynamic scapular/humeral head control

Coordination

Kinetic chain/core stability

Motor control

Movement/muscle patterns

Muscle activation/recruitment

Proprioception

Scapulo-humeral alignment

**Shoulder muscle strengthening**

Shoulder muscles:

Deltoids

Glenohumeral muscles

Rotator cuff

Scapular stabilizers

Scapulothoracic muscles

Where defined in text

Endurance

Hypertrophy

Loading

Isometric

Isotonic

Plyometrics

Resistive/resisted weights

Strengthening (tubing/Theraband/resistance bands/elastic bands/dumbbells/pulleys)

**Static posture correction**

Correcting shoulder positioning while the glenohumeral joint is static. Scapula setting prior to movement constitutes static posture correction.

Where defined in text

Faulty posture

Postural control of the scapula

Resting posture

Maintaining an exact scapular position
