# Supplementary material for: A systematic review to compare physiotherapy treatment programmes for atraumatic shoulder instability
Source: Shoulder Elbow. 2022 Feb 18;15(4):448–60. doi: 10.1177/17585732221080730 (PMC10395403; doi:10.1177/17585732221080730)
Supplement: sj-docx-4-sel-10.1177_17585732221080730 - Supplemental material for A systematic review to compare physiotherapy treatment programmes for atraumatic shoulder instability [file sj-docx-4-sel-10.1177_17585732221080730.docx]

| **Supplementary material 4** Outcomes from the included studies | | | | | | | | | | | | | | | | | | | | | | | | | | | | | | |  |  |  |
| --- | --- | --- | --- | --- | --- | --- | --- | --- | --- | --- | --- | --- | --- | --- | --- | --- | --- | --- | --- | --- | --- | --- | --- | --- | --- | --- | --- | --- | --- | --- | --- | --- | --- |
| Study title |  | Outcome |  |  | Baseline results | |  | Results timepoint A | | |  | Results timepoint B | | | |  | | Results timepoint C | | | | |  | | Results timepoint D | | | | | |  |  |  |
|  |  |  |  |  | Mean/  score | SD (or other variance) |  | Mean/  score | SD (or other variance) | Significance |  | Mean/  score | SD (or other variance) | Significance |  | | Mean/  score | | SD (or other variance) | | Significance | | |  | | Mean/  score | SD (or other variance) | | | Significance | | |  |
| Bateman et al. (2019) |  |  |  |  | Baseline |  |  | Post-treatment | | |  |  |  |  | |  | |  | |  | |  |  | |  | | |  |  | |  |  |  |
|  |  | OSIS | |  | 38 | 8.19 |  | 21.96 | 7.03 | p<0.001 |  |  |  |  | |  | |  | |  | |  |  | |  | | |  |  | |  |  |  |
|  |  | WOSI | |  | 45.10% | 18 |  | 85.81% | 16.33 | p<0.001 |  |  |  |  | |  | |  | |  | |  |  | |  | | |  |  | |  |  |  |
| Blacknall et al. (2014) |  |  |  |  | Baseline |  |  | Discharge | | |  |  |  |  | |  | |  | |  | |  |  | |  | | |  |  | |  |  |  |
|  |  | OSIS | |  | 22.70 | 8.8 |  | 40.9 | 6.7 | p<0.000 |  |  |  |  | |  | |  | |  | |  |  | |  | | |  |  | |  |  |  |
|  |  |  |  |  |  |  |  |  |  |  |  |  |  |  | |  | |  | |  | |  |  | |  | | |  |  | |  |  |  |
|  |  | WOSI | |  | 49.80% | 14.20% |  | 87% | 11.30% | p<0.000 |  |  |  |  | |  | |  | |  | |  |  | |  | | |  |  | |  |  |  |
| Ide et al. (2003) |  |  |  |  | Baseline |  |  | Post-treatment | | |  | Follow-up (mean 7 years, range 5-9 years) | | | | | | | | | | | | | | | | | | |  |  |  |
|  |  | MR Total | |  | 51.9 | 16.7 |  | 74.9 | 13.7 | p<0.001 |  |  |  |  | |  | |  | |  | |  |  | |  | | |  |  | |  |  |  |
|  |  | MR Function | |  | 27.1 |  |  | 39 |  | p<0.001 |  |  |  |  | |  | |  | |  | |  |  | |  | | |  |  | |  |  |  |
|  |  | MR Pain, Numbness | |  | 2.8 |  |  | 8.1 |  | p<0.001 |  |  |  |  | |  | |  | |  | |  |  | |  | | |  |  | |  |  |  |
|  |  | MR Stability | |  | 11.9 |  |  | 18.1 |  | p<0.001 |  |  |  |  | |  | |  | |  | |  |  | |  | | |  |  | |  |  |  |
|  |  | MR Motion | |  | 9 |  |  | 10 |  | p<0.001 |  |  |  |  | |  | |  | |  | |  |  | |  | | |  |  | |  |  |  |
|  |  | MR Grading | |  | 59 fair, 14 poor |  |  | 12 excellent, 36 good, 24 fair, 1 poor | | |  | 7/62 shoulders demonstrated recurrence of signs and symptoms. Because of failure of conservative treatment, 3 patients underwent open capsular shift | | | | | | | | | | | | | | | | | | |  |  |  |
|  |  | IR peak torque | |  | 1.05 | 0.24 |  | 1.26 | 0.27 | p<0.05 |  |  |  |  | |  | |  | |  | |  |  | |  | | |  |  | |  |  |  |
|  |  | ER peak torque | |  | 1.33 | 0.33 |  | 1.84 | 0.39 | p<0.05 |  |  |  |  | |  | |  | |  | |  |  | |  | | |  |  | |  |  |  |
|  |  | IR/ER peak torque ratio | |  | 84.7 | 23 |  | 70.5 | 13.7 | p<0.05 |  |  |  |  | |  | |  | |  | |  |  | |  | | |  |  | |  |  |  |
| Kiss et al. (2001) |  |  |  |  | Baseline |  |  | Post-treatment | | |  |  |  |  | |  | |  | |  | |  |  | |  | | |  |  | |  |  |  |
|  |  | Constant | |  |  |  |  | 76 | 16 |  |  |  |  |  | |  | |  | |  | |  |  | |  | | |  |  | |  |  |  |
|  |  | Age & gender-adjusted Constant | |  |  |  |  | 80 | 17 |  |  |  |  |  | |  | |  | |  | |  |  | |  | | |  |  | |  |  |  |
|  |  | Rowe | |  |  |  |  | 50 | 29 |  |  |  |  |  | |  | |  | |  | |  |  | |  | | |  |  | |  |  |  |
|  |  | Subjective Shoulder Rating | |  |  |  |  | 79 | 14 |  |  |  |  |  | |  | |  | |  | |  |  | |  | | |  |  | |  |  |  |
|  |  | Satisfaction | |  |  |  |  | 2.23 | 0.76 |  |  |  |  |  | |  | |  | |  | |  |  | |  | | |  |  | |  |  |  |
| Merolla et al. (2014) |  |  |  |  | Baseline |  |  | 3 months | | |  | 6 months | | | |  | | 12 months | | | | |  | | 24 months | | | | | |  |  |  |
|  |  | Flexion | |  | 98 | 15 |  | 160 | 8 | p=0.0052 |  | 171 | 6 | p=0.001 | |  | | 171 | | 5 | | p=0.001 |  | | 171 | | | 9 | p=0.002 | |  |  |  |
|  |  | Abduction | |  | 80 | 10 |  | 150 | 11 | p=0.0061 |  | 170 | 8 | p=0.001 | |  | | 170 | | 4 | | p<0.01 |  | | 170 | | | 7 | p<0.01 | |  |  |  |
|  |  | Internal rotation | |  | 90 | 10 |  | 90 | 10 | p>0.05 |  | 90 | 10 | p>0.05 | |  | | 90 | | 10 | | p>0.05 |  | | 90 | | | 10 | p>0.05 | |  |  |  |
|  |  | External rotation | |  | 40 | 8 |  | 80 | 9 |  |  | 90 | 4 | p<0.01 | |  | | 90 | | 6 | | p<0.01 |  | | 90 | | | 6 | p<0.01 | |  |  |  |
|  |  | DASH | |  | 52 | 8.1 |  | 46 | 7 | p=0.0398 |  | 34.8 | 7 | p=0.0018 | |  | | 16.7 | | 5 | | p=0.0001 |  | | 7.1 | | | 3 | p=0.00001 | |  |  |  |
|  |  | SPADI | |  | 0.56 | 0.09 |  | 0.47 | 0.06 | p=0.0401 |  |  |  | p=0.009 | |  | |  | |  | | p=0.0001 |  | |  | | |  | p=0.00001 | |  |  |  |
|  |  | Modified Rowe | |  |  |  |  | Fair |  |  |  | Good |  |  | |  | | Good | |  | |  |  | | Good | | |  |  | |  |  |  |
|  |  | PGA | |  |  |  |  | "Well" | mean 3.1; SD 1.1 | |  | Well |  |  | |  | | Well | |  | |  |  | | Well | | |  |  | |  |  |  |
| Misamore et al. (2005) |  |  |  |  |  |  |  | 2 year |  |  |  | 8 year |  |  | |  | |  | |  | |  |  | |  | | |  |  | |  |  |  |
|  |  | Pain relief | |  |  |  |  | 20/39 good or excellent | | |  | 23/36 good or excellent | | | |  | |  | |  | |  |  | |  | | |  |  | |  |  |  |
|  |  | Stability | |  |  |  |  | 21/39 good or excellent | | |  | 17/36 good or excellent | | | |  | |  | |  | |  |  | |  | | |  |  | |  |  |  |
|  |  | Limitations | |  |  |  |  | 12/39 return to sport | | |  | 7/36 given up all sports, 9/36 lifestyle or employment changes due to instability | | | |  | |  | |  | |  |  | |  | | |  |  | |  |  |  |
|  |  | Overall status | |  |  |  |  | 28/39 much better | | |  | 20/36 much better | | | |  | |  | |  | |  |  | |  | | |  |  | |  |  |  |
|  |  | Modified Rowe instiability score | |  |  |  |  |  |  |  |  | 5/36 excellent, 12/36 good | | | |  | |  | |  | |  |  | |  | | |  |  | |  |  |  |
| Scott et al. (2019) |  |  |  |  | Baseline |  |  | Post-treatment | | |  |  |  |  | |  | |  | |  | |  |  | |  | | |  |  | |  |  |  |
|  |  | OSIS | |  | median 21 | range 2-47 |  | 39 (median) | range 11-47 | p<0.001 |  |  |  |  | |  | |  | |  | |  |  | |  | | |  |  | |  |  |  |
|  |  | WOSI | |  | median 1117 | range 306-2028 |  | 485 (median) | range 0-1569 | p<0.001 |  |  |  |  | |  | |  | |  | |  |  | |  | | |  |  | |  |  |  |
| Takwale et al. (2000) |  |  |  |  | Baseline |  |  | Post-treatment | | |  |  |  |  | |  | |  | |  | |  |  | |  | | |  |  | |  |  |  |
|  |  | Visual analogue scale | |  | 2.93 | 1.29 |  | 8.07 | 1.56 |  |  |  |  |  | |  | |  | |  | |  |  | |  | | |  |  | |  |  |  |
|  |  | Practitioner-graded functional outcome | |  |  |  |  | 31 excellent, 21 good, 6 poor (shoulders) | | |  |  |  |  | |  | |  | |  | |  |  | |  | | |  |  | |  |  |  |
| Warby et al. (2018) |  |  |  |  | Baseline |  |  | Week 6 |  |  |  | Week 12 | | | |  | | Week 24 | |  | |  |  | |  | | |  |  | |  |  |  |
|  |  | MISS | W |  | 47.6 | 16.8 |  | 60.1 | 14.4 | p=0.793 (between-group), significant within-group differences vs baseline |  | 74.4 | 17.6 | p=0.064 (between-group), significant within-group differences vs baseline | |  | | 78.8 | | 13.1 | | p=0.002 (between-group), significant within-group differences vs baseline |  | |  | | |  |  | |  |  |  |
|  |  |  | R |  | 48.7 | 15.3 |  | 59.9 | 24.4 |  |  | 67.8 | 20.7 |  |  |  | | 66.6 | | 21.4 | |  |  | |  | | |  |  | |  |  |  |
|  |  | WOSI | W |  | 37.9 | 17.5 |  | 54 | 20.5 | p=0.667 (between-group), significant within-group differences vs baseline |  | 71.4 | 18.5 | p=0.018 (between-group), significant within-group differences vs baseline | |  | | 72.8 | | 15.7 | | p=0.008 (between-group), significant within-group differences vs baseline |  | |  | | |  |  | |  |  |  |
|  |  |  | R |  | 41.8 | 16 |  | 56.1 | 24.2 |  |  | 65.4 | 23.2 |  |  |  | | 66.7 | | 22.5 | |  |  | |  | | |  |  | |  |  |  |
|  |  | Orebro Musculoskeletal Pain Questionnaire | W |  | 93.7 | 31.9 |  | 73.8 | 23.8 | p=0.517 (between-group) |  | 59.3 | 24.5 | p=0.119 (between-group) | |  | | 59.5 | | 24.7 | | p=0.053 (between-group) |  | |  | | |  |  | |  |  |  |
|  |  |  | R |  | 89.3 | 25.1 |  | 73.3 | 7 |  |  | 64.9 | 29.8 |  |  |  | | 67.1 | | 30.3 | |  |  | |  | | |  |  | |  |  |  |
|  |  | Pain score | W |  | 5.6 | 2.1 |  | 3.2 | 2.1 | p=0.240 (between-group) |  | 2.7 | 2 | p=0.121 (between-group) | |  | | 1.9 | | 1.6 | | p=0.003 (between-group) |  | |  | | |  |  | |  |  |  |
|  |  |  | R |  | 4.4 | 2.4 |  | 2.9 | 2.3 |  |  | 2.4 | 2.3 |  |  |  | | 2.5 | | 2.1 | |  |  | |  | | |  |  | |  |  |  |
|  |  | Global Rating of Change | W |  |  |  |  | 2 (median) | 2-3(IQR) | p=0.778 (between-group) |  | 2 (median) | 2-2 (IQR) | p=0.738 (between-group) | |  | | 2 (median) | | 2-2 (IQR) | | p=0.239 (between-group) |  | |  | | |  |  | |  |  |  |
|  |  |  | R |  |  |  |  | 2 (median) | 2-3(IQR) |  |  | 2 (median) | 2-2 (IQR) |  |  |  | | 2 (median) | | 2-3 (IQR) | |  |  | |  | | |  |  | |  |  |  |
|  |  | Patient Satisfaction with Physiotherapy Care Score | W |  |  |  |  | 1 (median) | 1-1 (IQR) | p=0.231 (between-group) |  | 1 (median) | 1-1 (IQR) | p=0.215 (between-group) | |  | | 1 (median) | | 1-1 (IQR) | | p=0.385 (between-group) |  | |  | | |  |  | |  |  |  |
|  |  |  | R |  |  |  |  | 1 (median) | 1-2 (IQR) |  |  | 1 (median) | 1-2 (IQR) |  |  |  | | 1 (median) | | 1-2 (IQR) | |  |  | |  | | |  |  | |  |  |  |
|  |  | Patient Satisfaction with Physiotherapy Result Score | W |  |  |  |  | 1 (median) | 1-2 (IQR) | p=0.285 (between-group) |  | 1 (median) | 1-1.5 (IQR) | p=0.124 (between-group) | |  | | 1 (median) | | 1-2 (IQR) | | p=0.217 (between-group) |  | |  | | |  |  | |  |  |  |
|  |  |  | R |  |  |  |  | 2 (median) | 1-2 (IQR) |  |  | 1 (median) | 1-2 (IQR) |  |  |  | | 2 (median | | 1-2 (IQR) | |  |  | |  | | |  |  | |  |  |  |
|  |  | Incidence complete glenohumeral dislocation | W |  |  |  |  | 1 | 5.90% | p=0.624 (between-group) |  | 1 | 5.90% | p=0.436 (between-group) | |  | | 2 | | 11.80% | | p=0.593 (between-group) |  | |  | | |  |  | |  |  |  |
|  |  |  | R |  |  |  |  | 3 | 13% |  |  | 0 | 0% |  |  |  | | 1 | | 5.30% | |  |  | |  | | |  |  | |  |  |  |
|  |  | MS; ER at 0 | W |  | 6.28 | 2.2 |  |  |  |  |  | 7.4 | 1.8 | p=0.319 (between-group) | |  | |  | |  | |  |  | |  | | |  |  | |  |  |  |
|  |  |  | R |  | 6.7 | 2.1 |  |  |  |  |  | 8.5 | 2.4 |  |  |  | |  | |  | |  |  | |  | | |  |  | |  |  |  |
|  |  | MS; IR at 0 | W |  | 7.5 | 2.3 |  |  |  |  |  | 8.7 | 2.3 | p=0.257 (between-group) | |  | |  | |  | |  |  | |  | | |  |  | |  |  |  |
|  |  |  | R |  | 7.7 | 2.4 |  |  |  |  |  | 10.3 | 4.5 |  |  |  | |  | |  | |  |  | |  | | |  |  | |  |  |  |
|  |  | MS; Extension | W |  | 8.4 | 3 |  |  |  |  |  | 10.7 | 3.5 | p=0.704 (between-group) | |  | |  | |  | |  |  | |  | | |  |  | |  |  |  |
|  |  |  | R |  | 8.8 | 2.7 |  |  |  |  |  | 11.3 | 4.4 |  |  |  | |  | |  | |  |  | |  | | |  |  | |  |  |  |
|  |  | MS; Abduction | W |  | 8.8 | 3.4 |  |  |  |  |  | 11.1 | 3.4 | p=0.383 (between-group) | |  | |  | |  | |  |  | |  | | |  |  | |  |  |  |
|  |  |  | R |  | 9.9 | 3.7 |  |  |  |  |  | 11.5 | 3.7 |  |  |  | |  | |  | |  |  | |  | | |  |  | |  |  |  |
|  |  | MS; ER at 90 | W |  | 3.5 | 1.2 |  |  |  |  |  | 5.2 | 1.7 | p=0.174 (between-group) | |  | |  | |  | |  |  | |  | | |  |  | |  |  |  |
|  |  |  | R |  | 3.9 | 1.1 |  |  |  |  |  | 5.1 | 1.9 |  |  |  | |  | |  | |  |  | |  | | |  |  | |  |  |  |
|  |  | MS; IR at 90 | W |  | 5 | 2.1 |  |  |  |  |  | 5.9 | 1.5 | p=0.097 (between-group) | |  | |  | |  | |  |  | |  | | |  |  | |  |  |  |
|  |  |  | R |  | 4.5 | 1.3 |  |  |  |  |  | 6.6 | 2.4 |  |  |  | |  | |  | |  |  | |  | | |  |  | |  |  |  |
|  |  | MS; Empty can | W |  | 4.5 | 1.9 |  |  |  |  |  | 5.8 | 1.6 | p=0.134 (between-group) | |  | |  | |  | |  |  | |  | | |  |  | |  |  |  |
|  |  |  | R |  | 4.9 | 2.1 |  |  |  |  |  | 5.7 | 2.2 |  |  |  | |  | |  | |  |  | |  | | |  |  | |  |  |  |
|  |  | MS; Long lever flexion | W |  | 3.9 | 1.5 |  |  |  |  |  | 5.3 | 1.4 | p=0.617 (between-group) | |  | |  | |  | |  |  | |  | | |  |  | |  |  |  |
|  |  |  | R |  | 4.6 | 1.7 |  |  |  |  |  | 5.8 | 2.5 |  |  |  | |  | |  | |  |  | |  | | |  |  | |  |  |  |
|  |  | MS; Belly press | W |  | 5.3 | 1.7 |  |  |  |  |  | 6.7 | 1.6 | p=0.730 (between-group) | |  | |  | |  | |  |  | |  | | |  |  | |  |  |  |
|  |  |  | R |  | 5.9 | 1.9 |  |  |  |  |  | 7.4 | 2 |  |  |  | |  | |  | |  |  | |  | | |  |  | |  |  |  |
|  |  | MS; Upward rotation | W |  | 19 | 7.7 |  |  |  |  |  | 23.3 | 5.7 | p=0.442 (between-group) | |  | |  | |  | |  |  | |  | | |  |  | |  |  |  |
|  |  |  | R |  | 19.9 | 6.7 |  |  |  |  |  | 23.5 | 5.9 |  |  |  | |  | |  | |  |  | |  | | |  |  | |  |  |  |
|  |  | MS; Short lever flexion | W |  | 9.2 | 3.5 |  |  |  |  |  | 13.5 | 3.6 | p=0.001 (between-group) | |  | |  | |  | |  |  | |  | | |  |  | |  |  |  |
|  |  |  | R |  | 11.2 | 4.9 |  |  |  |  |  | 12.9 | 4.6 |  |  |  | |  | |  | |  |  | |  | | |  |  | |  |  |  |
|  |  | SUR; rest | W |  | 3 | 5.6 |  |  |  |  |  | 8.5 | 6.4 | p=0.471 (between-group) | |  | |  | |  | |  |  | |  | | |  |  | |  |  |  |
|  |  |  | R |  | -0.7 | 5.2 |  |  |  |  |  | 3.5 | 6.8 |  |  |  | |  | |  | |  |  | |  | | |  |  | |  |  |  |
|  |  | SUR; 30 degrees GH abduction | W |  | 8.3 | 6.3 |  |  |  |  |  | 13.7 | 7 | p=0.875 (between-group) | |  | |  | |  | |  |  | |  | | |  |  | |  |  |  |
|  |  |  | R |  | 3.1 | 8.8 |  |  |  |  |  | 9.2 | 9.6 |  |  |  | |  | |  | |  |  | |  | | |  |  | |  |  |  |
|  |  | SUR; 45 | W |  | 13.1 | 7.4 |  |  |  |  |  | 18.7 | 8.1 | p=0.869 (between-group) | |  | |  | |  | |  |  | |  | | |  |  | |  |  |  |
|  |  |  | R |  | 8.3 | 10.5 |  |  |  |  |  | 14.7 | 10.2 |  |  |  | |  | |  | |  |  | |  | | |  |  | |  |  |  |
|  |  | SUR; 60 | W |  | 18.4 | 8.8 |  |  |  |  |  | 23.9 | 8.9 | p=0.978 (between-group) | |  | |  | |  | |  |  | |  | | |  |  | |  |  |  |
|  |  |  | R |  | 13.9 | 8.4 |  |  |  |  |  | 20.3 | 11.3 |  |  |  | |  | |  | |  |  | |  | | |  |  | |  |  |  |
|  |  | SUR; 90 | W |  | 30.22 | 11 |  |  |  |  |  | 33.9 | 9.8 | p=0.473 (between-group) | |  | |  | |  | |  |  | |  | | |  |  | |  |  |  |
|  |  |  | R |  | 25.7 | 9.5 |  |  |  |  |  | 32.3 | 12.2 |  |  |  | |  | |  | |  |  | |  | | |  |  | |  |  |  |
|  |  | SUR; 120 | W |  | 39.2 | 9.4 |  |  |  |  |  | 44.4 | 10 | p=0.546 (between-group) | |  | |  | |  | |  |  | |  | | |  |  | |  |  |  |
|  |  |  | R |  | 36.9 | 10.8 |  |  |  |  |  | 42.7 | 10 |  |  |  | |  | |  | |  |  | |  | | |  |  | |  |  |  |
|  |  | SUR; 135 | W |  | 45.4 | 7.33 |  |  |  |  |  | 50.9 | 7.9 | p=0.286 (between-group) | |  | |  | |  | |  |  | |  | | |  |  | |  |  |  |
|  |  |  | R |  | 44.6 | 8.7 |  |  |  |  |  | 48.8 | 9.6 |  |  |  | |  | |  | |  |  | |  | | |  |  | |  |  |  |
|  |  | SUR; EOR | W |  | 57.1 | 9.4 |  |  |  |  |  | 62.5 | 6.6 | p=0.159 (between-group) | |  | |  | |  | |  |  | |  | | |  |  | |  |  |  |
|  |  |  | R |  | 57.2 | 11.5 |  |  |  |  |  | 60.4 | 9.4 |  |  |  | |  | |  | |  |  | |  | | |  |  | |  |  |  |
|  |  | Scapular co-ordinates at rest inferior angle x | W |  | 8 | 1.7 |  |  |  |  |  | 8 | 1.6 | p=0.131 (between-group) | |  | |  | |  | |  |  | |  | | |  |  | |  |  |  |
|  |  |  | R |  | 7.6 | 1.6 |  |  |  |  |  | 8.2 | 2.3 |  |  |  | |  | |  | |  |  | |  | | |  |  | |  |  |  |
|  |  | SCr inf y | W |  | 18.7 | 1.4 |  |  |  |  |  | 18.7 | 1.2 | p=0.243 (between-group) | |  | |  | |  | |  |  | |  | | |  |  | |  |  |  |
|  |  |  | R |  | 18.8 | 1.6 |  |  |  |  |  | 18 | 2.8 |  |  |  | |  | |  | |  |  | |  | | |  |  | |  |  |  |
|  |  | SCr medial x | W |  | 8.2 | 1.9 |  |  |  |  |  | 8 | 1.1 | p=0.090 (between-group) | |  | |  | |  | |  |  | |  | | |  |  | |  |  |  |
|  |  |  | R |  | 8.4 | 1.2 |  |  |  |  |  | 8.2 | 1.1 |  |  |  | |  | |  | |  |  | |  | | |  |  | |  |  |  |
|  |  | SCr med y | W |  | 7.5 | 1.4 |  |  |  |  |  | 7.6 | 1.6 | p=0.592 (between-group) | |  | |  | |  | |  |  | |  | | |  |  | |  |  |  |
|  |  |  | R |  | 7.5 | 1.2 |  |  |  |  |  | 7.2 | 1.4 |  |  |  | |  | |  | |  |  | |  | | |  |  | |  |  |  |
|  |  | SCr ACJ x | W |  | 17.2 | 1.5 |  |  |  |  |  | 16.6 | 1.4 | p=0.055 (between-group) | |  | |  | |  | |  |  | |  | | |  |  | |  |  |  |
|  |  |  | R |  | 17.3 | 2.1 |  |  |  |  |  | 17.5 | 1.8 |  |  |  | |  | |  | |  |  | |  | | |  |  | |  |  |  |
|  |  | SCr ACJ y | W |  | 0.9 | 1.5 |  |  |  |  |  | 1 | 1.2 | p=0.009 (between-group) | |  | |  | |  | |  |  | |  | | |  |  | |  |  |  |
|  |  |  | R |  | 1.2 | 1 |  |  |  |  |  | 0.9 | 0.9 |  |  |  | |  | |  | |  |  | |  | | |  |  | |  |  |  |
|  |  | SC at 90deg abd inf x | W |  | 12.9 | 1.5 |  |  |  |  |  | 13.9 | 2 | p=0.256 (between-group) | |  | |  | |  | |  |  | |  | | |  |  | |  |  |  |
|  |  |  | R |  | 13.9 | 2.7 |  |  |  |  |  | 14.3 | 2.3 |  |  |  | |  | |  | |  |  | |  | | |  |  | |  |  |  |
|  |  | SC90 inf y | W |  | 18.5 | 2.1 |  |  |  |  |  | 19.1 | 1.6 | p=0.033 (between-group) | |  | |  | |  | |  |  | |  | | |  |  | |  |  |  |
|  |  |  | R |  | 18.7 | 2.1 |  |  |  |  |  | 18 | 1.4 |  |  |  | |  | |  | |  |  | |  | | |  |  | |  |  |  |
|  |  | SC90 med x | W |  | 8.8 | 2.3 |  |  |  |  |  | 7.1 | 2.9 | p=0.778 (between-group) | |  | |  | |  | |  |  | |  | | |  |  | |  |  |  |
|  |  |  | R |  | 8.5 | 1.9 |  |  |  |  |  | 8.5 | 1.7 |  |  |  | |  | |  | |  |  | |  | | |  |  | |  |  |  |
|  |  | SC90 med y | W |  | 8.3 | 1.6 |  |  |  |  |  | 8.5 | 1.9 | p=0.164 (between-group) | |  | |  | |  | |  |  | |  | | |  |  | |  |  |  |
|  |  |  | R |  | 8.5 | 2.2 |  |  |  |  |  | 8.2 | 2 |  |  |  | |  | |  | |  |  | |  | | |  |  | |  |  |  |
|  |  | SC90 ACJ x | W |  | 14.1 | 1.7 |  |  |  |  |  | 13.6 | 1.5 | p=0.140 (between-group) | |  | |  | |  | |  |  | |  | | |  |  | |  |  |  |
|  |  |  | R |  | 14.1 | 2.2 |  |  |  |  |  | 14.2 | 2.6 |  |  |  | |  | |  | |  |  | |  | | |  |  | |  |  |  |
|  |  | SC90 ACJ y | W |  | -1 | 0.9 |  |  |  |  |  | -0.9 | 0.8 | p=0.391 (between-group) | |  | |  | |  | |  |  | |  | | |  |  | |  |  |  |
|  |  |  | R |  | -1.1 | 1 |  |  |  |  |  | -1.3 | 0.8 |  |  |  | |  | |  | |  |  | |  | | |  |  | |  |  |  |
|  |  | SC at end range of motion inf x | W |  | 16.9 | 2.7 |  |  |  |  |  | 17.88 | 1.5 | p=0.462 (between-group) | |  | |  | |  | |  |  | |  | | |  |  | |  |  |  |
|  |  |  | R |  | 18.1 | 3 |  |  |  |  |  | 18.6 | 2.6 |  |  |  | |  | |  | |  |  | |  | | |  |  | |  |  |  |
|  |  | SCer inf y | W |  | 16.8 | 1.7 |  |  |  |  |  | 16.8 | 3.2 | p=0.973 (between-group) | |  | |  | |  | |  |  | |  | | |  |  | |  |  |  |
|  |  |  | R |  | 17 | 1.6 |  |  |  |  |  | 17 | 1.5 |  |  |  | |  | |  | |  |  | |  | | |  |  | |  |  |  |
|  |  | SCer med x | W |  | 10.1 | 2.4 |  |  |  |  |  | 10.1 | 2.2 | p=0.371 (between-group) | |  | |  | |  | |  |  | |  | | |  |  | |  |  |  |
|  |  |  | R |  | 10.5 | 2.8 |  |  |  |  |  | 10.1 | 2.4 |  |  |  | |  | |  | |  |  | |  | | |  |  | |  |  |  |
|  |  | SCer med y | W |  | 9.6 | 1.6 |  |  |  |  |  | 10.2 | 2 | p=0.997 (between-group) | |  | |  | |  | |  |  | |  | | |  |  | |  |  |  |
|  |  |  | R |  | 9.4 | 1.7 |  |  |  |  |  | 9.9 | 1.5 |  |  |  | |  | |  | |  |  | |  | | |  |  | |  |  |  |
|  |  | SCer ACJ x | W |  | 12.1 | 1.8 |  |  |  |  |  | 11.4 | 1.9 | p=0.141 (between-group) | |  | |  | |  | |  |  | |  | | |  |  | |  |  |  |
|  |  |  | R |  | 11.7 | 1.9 |  |  |  |  |  | 11.9 | 2.5 |  |  |  | |  | |  | |  |  | |  | | |  |  | |  |  |  |
|  |  | SCer ACJ y | W |  | -1.2 | 1.1 |  |  |  |  |  | -0.8 | 1.1 | p=0.079 (between-group) | |  | |  | |  | |  |  | |  | | |  |  | |  |  |  |
|  |  |  | R |  | -1.4 | 1.5 |  |  |  |  |  | -1.5 | 1.2 |  |  |  | |  | |  | |  |  | |  | | |  |  | |  |  |  |
| Watson et al. (2018) |  |  |  |  | Baseline |  |  | Post-treatment | | |  |  |  |  | |  | |  | |  | |  |  | |  | | |  |  | |  |  |  |
|  |  | WOSI | |  | 1264.63 | SD 327.95; range 465-1820; median 1285.0; IQR 520.0 |  | 482.23 | SD 252.11; range 105-1285; median 492.0; IQR 265.0 | p<0.001 |  |  |  |  | |  | |  | |  | |  |  | |  | | |  |  | |  |  |  |
|  |  | MISS | |  | 46.95 | SD 15.75; range 19-81; median 46.5; IQR 23.3 |  | 76.32 | SD 12.20; range 35.5-92; median 79.5; IQR 13.2 | p<0.001 |  |  |  |  | |  | |  | |  | |  |  | |  | | |  |  | |  |  |  |
|  |  | OSIS | |  | 35.76 | SD 8.59; range 20-53; median 36.0; IQR 14 |  | 20.67 | SD 6.97; range 13-40; median 19.0; IQR 8.0 | p<0.001 |  |  |  |  | |  | |  | |  | |  |  | |  | | |  |  | |  |  |  |
|  |  | Numerical Rating Scale | |  | 4 | SD 1.8; range 0-7.5; median 4.0; IQR 2.0 |  | 1.6 | SD .93; range 0-3; median 1.5; IQR 1.0 | p<0.001 |  |  |  |  | |  | |  | |  | |  |  | |  | | |  |  | |  |  |  |
|  |  | SUR; rest | |  | 5.3 | 8.8 |  | 12.3 | 5.5 | p<0.001 |  |  |  |  | |  | |  | |  | |  |  | |  | | |  |  | |  |  |  |
|  |  | SUR; 30 degrees GH abduction | |  | 11.2 | 10.4 |  | 17.6 | 5.9 | p<0.001 |  |  |  |  | |  | |  | |  | |  |  | |  | | |  |  | |  |  |  |
|  |  | SUR; 45 | |  | 18.1 | 11.9 |  | 23.8 | 6.5 | 0.001 |  |  |  |  | |  | |  | |  | |  |  | |  | | |  |  | |  |  |  |
|  |  | SUR; 60 | |  | 26.8 | 12.7 |  | 31.5 | 8.3 | 0.002 |  |  |  |  | |  | |  | |  | |  |  | |  | | |  |  | |  |  |  |
|  |  | SUR; 90 | |  | 41.9 | 11.4 |  | 42.9 | 8.4 | 0.232 |  |  |  |  | |  | |  | |  | |  |  | |  | | |  |  | |  |  |  |
|  |  | SUR; 120 | |  | 55.5 | 9.6 |  | 54.6 | 7.5 | 0.715 |  |  |  |  | |  | |  | |  | |  |  | |  | | |  |  | |  |  |  |
|  |  | SUR; 135 | |  | 59.5 | 9.3 |  | 60.5 | 6.5 | 0.534 |  |  |  |  | |  | |  | |  | |  |  | |  | | |  |  | |  |  |  |
|  |  | SUR; EOR | |  | 68.9 | 9.2 |  | 67.1 | 5.7 | 0.082 |  |  |  |  | |  | |  | |  | |  |  | |  | | |  |  | |  |  |  |
|  |  | Scapular coordinates | |  |  |  |  | No significant difference found for any measure |  |  |  |  |  |  | |  | |  | |  | |  |  | |  | | |  |  | |  |  |  |
|  |  | MS; empty can | |  | 53.6 (median 50.6; IQR 24.2) | 22.5 |  | 68.6 (median 68.2; IQR 29.1) | 22.5 | p<0.001 |  |  |  |  | |  | |  | |  | |  |  | |  | | |  |  | |  |  |  |
|  |  | MS; ER 0 degrees | |  | 84.4 (median 83.6; IQR 41.4) | 34.5 |  | 101 (median 101; IQR 37) | 25.4 | p<0.001 |  |  |  |  | |  | |  | |  | |  |  | |  | | |  |  | |  |  |  |
|  |  | MS; ER 90 degrees | |  | 52.1 (median 48.4; IQR 24.2) | 23.7 |  | 79.4 (median 72.6; IQR 30.3) | 27.7 | p<0.001 |  |  |  |  | |  | |  | |  | |  |  | |  | | |  |  | |  |  |  |
|  |  | MS; Lift off HBB | |  | 47.3 (median 42.9; IQR 27.5) | 22 |  | 59.1 (medican 53.5; IQR 28.8) | 27 | p<0.001 |  |  |  |  | |  | |  | |  | |  |  | |  | | |  |  | |  |  |  |
|  |  | MS; IR 90 degrees | |  | 81 (median 68.2; IQR 52.4) | 41.6 |  | 122.2 (median 121; IQR 44.3) | 45.9 | p<0.001 |  |  |  |  | |  | |  | |  | |  |  | |  | | |  |  | |  |  |  |
|  |  | MS; biceps (speed test) | |  | 58.6 (median 59.4; IQR 39.8) | 27 |  | 68.8 (median 66; IQR 29.8) | 24.4 | p<0.001 |  |  |  |  | |  | |  | |  | |  |  | |  | | |  |  | |  |  |  |
|  |  | MS; anterior deltoid (short lever flexion) | |  | 137.6 (median 125; IQR 59) | 65.3 |  | 168.8 (median 154.5; IQR 62.5) | 58 | p<0.001 |  |  |  |  | |  | |  | |  | |  |  | |  | | |  |  | |  |  |  |
|  |  | MS; middle deltoid (abduction) | |  | 98.9 (median 97; IQR 48.4) | 40.7 |  | 120.9 (median 112; IQR 42.3) | 37.9 | p<0.001 |  |  |  |  | |  | |  | |  | |  |  | |  | | |  |  | |  |  |  |
|  |  | MS; posterior deltoid (extension) | |  | 154.4 (median 143; IQR 111.8) | 68 |  | 212.4 (median 205.5; IQR 77.4) | 52.8 | p<0.001 |  |  |  |  | |  | |  | |  | |  |  | |  | | |  |  | |  |  |  |
|  |  | MS; shrug | |  | 196.4 (median 173; IQR 66) | 61.4 |  | 288.8 (median 281; IQR 90) | 64.8 | p<0.001 |  |  |  |  | |  | |  | |  | |  |  | |  | | |  |  | |  |  |  |
| DASH; Disability of the Arm, Shoulder and Hand score, EOR; End of Range, ER; External Rotation, GH; Glenohumeral, IQR; Interquartile Range, IR; Internal Rotation, MISS; Melbroune Instability Shoulder Score, MR; Modified Rowe, MS; Muscle Strength, OSIS; Oxford Shoulder Instability Score, PGA; Patient Global Assessment, SD; Standard Deviation, SPADI; Shoulder Pain and Disability Index, SUR; Scapula Upward Rotation, WOSI; Western Ontario Shoulder Index | | | | | | | | | | | | | | | | | | | | | | | | | | | | | | |  |  |  |
